# Minimally Invasive Ventriculo-Cholecystic Shunt Placement Utilizing Combined Ultrasound and Fluoroscopic Guidance

**DOI:** 10.7759/cureus.31830

**Published:** 2022-11-23

**Authors:** Oliver D Mrowczynski, Sonia S Majid, Frank C Lynch, Scott D Simon

**Affiliations:** 1 Neurosurgery, Penn State Health Milton S. Hershey Medical Center, Hershey, USA; 2 Interventional Radiology, Penn State Health Milton S. Hershey Medical Center, Hershey, USA; 3 Neurological Surgery, Penn State Health Milton S. Hershey Medical Center, Hershey, USA

**Keywords:** hydrocephalus, shunt, ventriculo-gall bladder, ventriculo-biliary, ventriculo-cholecystic

## Abstract

For the management of hydrocephalus with ventricular cerebrospinal fluid (CSF) shunting, multiple therapeutic options are available. Among these routes, the most commonly used are ventriculo-peritoneal, ventriculo-atrial, and ventriculo-pleural, while ventriculo-cholecystic is a less common option. Although ventriculo-peritoneal is accepted as the first option, ventriculo-cholecystic shunting may be performed in patients who are poor candidates for other routes of shunt placement. Open cholecystic shunt placement may be contraindicated in patients who have undergone previous surgeries or other comorbidities. Here, we present the case of a 25-year-old female with a complex medical history who presented with a posterior fossa intraparenchymal hemorrhage and subsequent hydrocephalus. She was unable to undergo a ventriculo-peritoneal, atrial, or pleural shunt placement, and thus, a cholecystic shunt placement was chosen. Due to a history of previous surgeries and comorbidities as well as a large volume of idiopathic and recurrent ascites, open placement was contraindicated in this patient. To the best of our knowledge, we present the first successful adult case of a minimally invasive ventriculo-cholecystic shunt placement under ultrasound and fluoroscopic guidance.

## Introduction

For the management of hydrocephalus with ventricular cerebrospinal fluid (CSF) shunting, multiple therapeutic options are available. The most common options include ventriculo-peritoneal, ventriculo-atrial, and ventriculo-pleural. However, ventriculo-cholecystic is less commonly utilized option. Morosanu et al. [1] described over 36 options for the placement of distal ventricular shunts [1]. Other distal access sites include atrial and pleural, with ventriculo-cholecystic being the last line route for distal access. Ventriculo-cholecystic placement was first described by Smith et al. [2] as an experimental trial. In the case of a ventriculo-peritoneal or ventriculo-pleural shunt, the CSF is absorbed from the space to which it is drained, either through the peritoneum or through the pleural space [3]. The gallbladder’s main function is to secrete bile for gastrointestinal processes and is minimally involved in absorption. In a ventriculo-cholecystic shunt, the CSF is mostly eliminated through the sphincter of Oddi relaxation and is subsequently released into the gastrointestinal tract [4].

To ensure the feasibility of ventriculo-cholecystic shunt placement, multiple parameters need to be considered, including gallbladder size and any pre-existing gallbladder pathology such as calculi. Thus, imaging techniques such as abdominal ultrasound and abdominal computed tomography (CT) are recommended [5]. Here, we present, to the best of our knowledge, the first reported adult case of a minimally invasive ventriculo-cholecystic shunt placement with ultrasound and fluoroscopic guidance.

## Case presentation

We present the case of a 25-year-old female with a very complex medical history of CLOVES (congenital lipomatous (fatty) overgrowth, vascular malformations, epidermal nevi, and scoliosis/skeletal/spinal anomalies) syndrome, Klippel-Trenaunay syndrome, multiple superior vena cava anomalies, venous clotting, cerebral palsy, autism, and so on. The patient presented to our medical center with a large left posterior fossa intraparenchymal hemorrhage (IPH). She underwent suboccipital craniectomy for IPH evacuation and external ventricular drain (EVD) placement.

Her course was complicated by abdominal compartment syndrome, requiring a bedside exploratory laparotomy and subsequent operations for total colectomy and ileostomy. She had ongoing respiratory failure, and consequently, a tracheostomy and a percutaneous endoscopic gastrostomy tube were placed. After multiple failed EVD weaning attempts and subsequent hydrocephalus, ventricular shunt placement was needed. Due to her history of multiple abdominal surgeries and her ascites status after colectomy, her candidacy for ventriculo-peritoneal shunt placement was considered poor. Because of her vascular anomalies, difficult anatomy, and extensive venous clotting, a ventriculo-atrial shunt was also not considered safe. Due to her history of scoliosis and anatomic abnormalities and since her lungs were atypically small and prone to infection with multiple bouts of pneumonia, a ventriculo-pleural shunt was not considered feasible. Hence, ventriculo-cholecystic shunt placement was deemed the best option. As the patient had multiple previous abdominal operations, the general surgery team felt that open cholecystostomy for shunt placement was relatively high risk.

**Figure 1:**
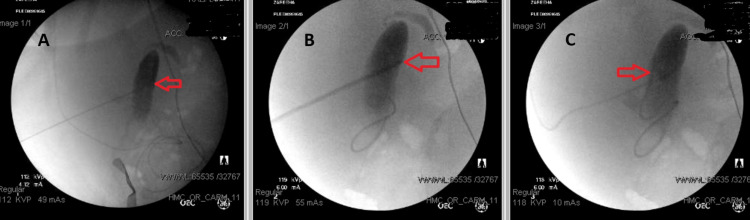
Fluoroscopic images of distal ventriculo-cholecystic shunt placement. A) Needle placement into the gallbladder, followed by contrast injection to confirm access to the gallbladder as well as the patency of the cystic duct to ensure that cerebrospinal fluid (CSF) had outflow (arrow points to distal needle tip in the gallbladder with subsequent contrast injection). B) Under fluoroscopic guidance and using a wire, the peel-away sheath was introduced into the gallbladder. The distal end of the shunt was then introduced into the gallbladder through the peel-away sheath, coiling approximately 10 cm around the body and the fundus of the gallbladder in a minimally invasive manner (arrow points to the tip of the peel-away sheath and indicates the insertion of the distal shunt catheter into the contrast-filled gallbladder). C) Removal of the peel-away sheath and final distal catheter placement in the gallbladder. Following the removal of the sheath, contrast injection through the more proximal shunt catheter was performed under fluoroscopic observation to confirm the opacification of the gallbladder (arrow).

We, thus, consulted with our interventional radiology colleagues, who advised us to consider the placement of the distal component of the shunt under fluoroscopic and ultrasound image guidance. We placed the proximal portion of the shunt using the standard neurosurgical technique. A C-shaped incision was made on the patient’s ride side Kocher’s point, 11 cm posterior from the glabella and 3 cm lateral from midline. After the incision, a burr hole was drilled at the same site. The dural leaflets were cut with blade number 11, and the leaflets were coagulated back. The proximal ventricular catheter was placed using EVD trajectories, aiming for the ipsilateral medial canthus and the ipsilateral tragus, and brisk CSF flow was noted. A neuro-endoscope was then placed inside the ventricular catheter, and the catheter was placed in the ipsilateral ventricle at the entrance of the foramen of Monroe under direct visualization. Subsequently, under minimally invasive ultrasound guidance, percutaneous access to the gallbladder body/neck through the transhepatic course was performed using an 18G INRAD needle (INRAD Inc., Kentwood MI), which is an approach typical for cholecystostomy tube placement. Contrast was injected using the needle under fluoroscopic guidance to document and confirm access to the gallbladder as well as the patency of the cystic duct. Over an Amplatz Extrastiff wire (Cook Medical, Bloomington IN), a 9 Fr 15 cm peel-away sheath (Galt Medical, Garland TX) was introduced into the gallbladder. Under fluoroscopic guidance, the distal end of the shunt was then introduced into the gallbladder through the peel-away sheath, coiling approximately 10 cm around the body and fundus of the gallbladder, ensuring minimal invasion. Following catheter placement and the removal of the peel-away sheath, contrast injection through the more proximal shunt catheter was performed under fluoroscopic observation to confirm opacification of the gallbladder (Figures 1A, 1B, and 1C). There were no intraoperative or postoperative complications during the procedure. Currently, the patient is in stable condition, and the shunt appears to be functioning well without any issues. She is still admitted to our center, pending rehabilitation placement one month after the shunt placement.

## Discussion

Hydrocephalus can be managed using multiple therapeutic options, with the most common being ventricular shunting. For ventricular shunting, over 30 options have been reported for the location of the distal placement of the shunt catheter [1]. The present case shows the successful management of a complex patient with hydrocephalus through the minimally invasive placement of a ventriculo-cholecystic shunt. This patient had numerous previous abdominal surgeries and subsequent ascites, as a result of which peritoneal placement was not feasible. She had atypical small lungs and was prone to infection, and thus, pleural placement was not possible. She also had difficult anatomy and a history of venous clotting, and thus, atrial placement was also not a feasible option. Hence, distal shunt placement into the gallbladder was considered.

The general technique of ventriculo-cholecystic placement involves placing the proximal component as described above and then planning a right subcostal incision for the distal component [6]. The gallbladder is found to be open, and after isolating the gallbladder, purse string sutures are placed at the dome. Then, a cholecystostomy is made in the dome of the gallbladder at the site of the purse string sutures, and the distal end of the shunt is then placed into the cholecystostomy [7]. The purse string sutures are then tightened to secure the distal catheter in the gallbladder. Different reports have indicated different lengths of the distal catheter placed into the gallbladder, ranging from 2 cm to 7 cm [8], however, there is no clear recommendation. In patients with multiple previous abdominal surgeries and other comorbidities, an open ventriculo-cholecystic shunt may not be feasible.

The patient had a history of multiple abdominal surgeries, and thus, the open placement of the distal shunt into the gallbladder was contraindicated. She, thus, underwent placement of a minimally invasive ventriculo-cholecystic shunt placement with ultrasound and fluoroscopic guidance. Regarding the type of shunt utilized, unitized shunts are not optimal because a contrast run is a helpful step to confirm proper shunt tubing placement. With unitized shunts, the shunt valve is pre-connected to the distal catheter by the manufacturer, and thus, the distal portion of the shunt catheter cannot be easily accessed for contrast injection. After performing the contrast run, the system should be flushed before being connected to CSF to wash out the contrast. Unlike an open ventriculo-cholecystic shunt where a cholecystostomy is performed and purse string sutures are used, in our minimally invasive procedure, no purse string sutures were needed, as the shunt tubing ran through the liver. This technique is one that surgeons who frequently perform ventricular shunting procedures can incorporate into their arsenal of the treatment of medically complex patients.

Complications of ventriculo-cholecystic shunt placement, specifically include things such as biliary ventriculitis, bile peritonitis, possibly cholecystitis or cholangitis, and gallbladder atony [2]. These can be evaluated using a hepatobiliary iminodiacetic acid scan or endoscopic retrograde cholangiopancreatography [4].

## Conclusions

Here, we present, to the best of our knowledge, the first reported successful adult case of minimally invasive ventriculo-cholecystic shunt placement with ultrasound and fluoroscopic guidance. We believe this to be a feasible technique for patients with hydrocephalus and who are poor candidates for other shunt placement options. Minimally invasive ventriculo-cholecystic shunt placement is a viable and effective option for ventricular shunting in medically complex patients.
